# Supplementary material for: Canine Caregivers: Paradoxical Challenges and Rewards
Source: Animals (Basel). 2022 Apr 21;12(9):1074. doi: 10.3390/ani12091074 (PMC9099636; doi:10.3390/ani12091074)
Supplement: Supplementary file 1 [file animals-12-01074-s001.zip › animals-1675203-SI.pdf]

Your experiences and perceptions related to your aging dogs are wanted. We are looking for dog guardians at least 18 years of age who are the primary caretaker of an aging dog and have owned this dog for at least 3 years.
If you meet these qualifications, we would like to ask you to please consider taking the following short anonymous survey.

Fine Print:
What is involved?
You will be asked to complete a series of questions as honestly as possible and there are no right or wrong answers. The questionnaire should take no more than 10 minutes to complete. Participation is entirely voluntary. You may quit at any time. This study has been approved by Colorado State University Research and Integrity Compliance and Review Board.

Are there any benefits or risks in my taking part?
There are no direct risks or benefits to completing the survey. The survey is voluntary and anonymous and you may stop the survey at any time by closing the window. Data from the survey will be used only for research and will hopefully be published in a journal.
Will my participation be confidential?
Yes, all participation will be confidential. The data will be anonymous and will contain no information that could lead to the identity of individuals. Anonymous data will be kept on a password protected computer.

What happens if I change my mind?
If you feel you do not wish to continue with the questionnaire, you can close the browser window.

Where can I get more information?
If you have questions about this research please contact Dr. Lori Kogan (Lori.Kogan@ColoState.EDU). Any questions about participant rights related to this survey can be directed to CSU IRB (ricro_irb@mail.colostate.edu) or 970 491-1655. Questions about the survey can be directed to Lori Kogan, PhD at lori.kogan@colostate.edu.

Please indicate if you would like to continue:

○ Yes

○ No

NOTE: If you have more than one dog, please answer the following survey questions for the dog whose name begins with the letter that comes first in the alphabet. For example, if you have a dog named Duffy and another dog named Roy, please answer the survey about Duffy, since “D” comes before “R” in the alphabet.

Do you have an aging dog?

- ○ Yes
- ○ No

How long have you owned your aging dog?

- ○ Less than 3 years
- ○ 3-5 years
- ○ 5-7 years
- ○ More than 7 years

Please answer the following questions about your aging dog:

	Strongly disagree	Somewhat disagree	Somewhat agree	Strongly agree
My dog means more to me than any of my friends	○	○	○	○
Quite often I confide in my dog.	○	○	○	○
I believe that dogs should have the same rights and privileges as family members.	○	○	○	○
I believe my dog is my best friend.	○	○	○	○
Quite often, my feelings toward people are affected by the way they react to my dog.	○	○	○	○
I love my dog because he/she is more loyal to me than most of the people in my life.	○	○	○	○
I enjoy showing other people pictures of my dog.	○	○	○	○
I think my dog is just a pet.	○	○	○	○
I love my dog because it never judges me.	○	○	○	○
Please select strongly disagree	○	○	○	○
My dog knows when I’m feeling	○	○	○	○

bad.				
I often talk to other people about my dog.	○	○	○	○
My dog understands me.	○	○	○	○
I believe that loving my dog helps me stay healthy.	○	○	○	○
Dogs deserve as much respect as humans do.	○	○	○	○
My dog and I have a very close relationship.	○	○	○	○
I would do anything to take care of my dog.	○	○	○	○
I play with my dog often.	○	○	○	○
I consider my dog to be a great companion.	○	○	○	○
My dog makes me feel happy.	○	○	○	○
I feel that my dog is part of my family.	○	○	○	○
I am <u>not</u> very attached to my dog.	○	○	○	○
Owning a dog adds to my happiness.	○	○	○	○
I consider my dog to be a friend.	○	○	○	○

Please indicate any changes in <u>your lifestyle</u> based on your dog's aging:

	A lot less	A little less	About the same	A little more	A lot more
My amount of exercise	○	○	○	○	○
How much I socialize	○	○	○	○	○
The amount of time I spend time with my dog	○	○	○	○	○
My travel in the car with my dog	○	○	○	○	○
My travel/trips (at least overnight) without my dog	○	○	○	○	○

Please indicate any changes <u>in your dog</u>, due to aging, for each of the following:

	Much less	Less	Same	More	Much more
His/her activity level	○	○	○	○	○
His/her restlessness	○	○	○	○	○
His/her mobility level	○	○	○	○	○
How calm/relaxed he/she is	○	○	○	○	○
How vocal he/she is	○	○	○	○	○
How affectionate he/she is	○	○	○	○	○
His/her maturity level	○	○	○	○	○
His/her ability to understand your feelings and know what you are thinking	○	○	○	○	○

Please indicate your agreement level with the following statements regarding your aging dog:

	Strongly disagree	Disagree	Neutral	Agree	Strongly agree
I tend to bend my dog-related rules more as my dog ages (i.e., I let my dog sleep on the couch or bed, I give treats more often)	○	○	○	○	○
I would like to be able to walk/run further with my dog than he/she can now walk	○	○	○	○	○
I feel guilty when I exercise or go for a walk and can no longer take my dog with me	○	○	○	○	○
There are times I resent the changes I have had to make in my daily schedule to care for my dog	○	○	○	○	○
I dread leaving my dog for any period because of his/her age	○	○	○	○	○
I find I am more protective of my dog as he/she ages	○	○	○	○	○
Caring for my aging dog gives me a sense of purpose	○	○	○	○	○
I often feel depressed or sad watching my aging dog	○	○	○	○	○
It is nice to have an aging dog because he/she is so calm and quiet	○	○	○	○	○
I enjoy the fact that my dog is more snuggly as he/she ages	○	○	○	○	○
I cherish the time I spend with my aging dog	○	○	○	○	○

I worry how the loss of my aging dog will affect me and my family	○	○	○	○	○
I am worried other dogs will accidently hurt my aging dog	○	○	○	○	○
I worry about my ability to afford veterinary care for my aging dog	○	○	○	○	○
I worry that the number of remaining days with my dog are limited	○	○	○	○	○

Please indicate your agreement level with the following medical aspects of your aging dog:

	Strongly disagree	Disagree	Neutral	Agree	Strongly agree
I worry a great deal about when my dog can no longer get around by him/herself	○	○	○	○	○
I have begun wondering when the right time will be to think about talking to my vet about medications or treatments to help my aging dog	○	○	○	○	○
I have begun wondering when the right time will be to think about euthanasia	○	○	○	○	○
I have had to spend a lot of money on caring for my dog as he/she ages	○	○	○	○	○
I give my dog age-related over-the-counter supplements (NOT CBD/cannabis) for age related changes	○	○	○	○	○
I give my aging dog CBD/ cannabis products for age related changes	○	○	○	○	○

Please indicate your agreement level with the following statements regarding support for the care of your aging dog:

	Strongly disagree	Disagree	Neutral	Agree	Strongly agree
I talk with friends or my family about my concerns related to my aging dog	○	○	○	○	○
I have talked to my vet about my concerns related to my aging dog	○	○	○	○	○
I feel my vet and I are a team when it comes to caring for my aging dog	○	○	○	○	○
I wish I had someone to talk to about my aging dog	○	○	○	○	○
My dog gives my life purpose, and I am worried about what I will do without him/her	○	○	○	○	○
I dread the day my dog is no longer with me	○	○	○	○	○
Please select the response "Disagree"	○	○	○	○	○
I feel my family and/or friends do not understand what is needed to care for an aging dog	○	○	○	○	○

Please answer the following questions based on how you feel about your aging dog:

	Never	Rarely	Sometimes	Often	Always
I feel myself longing and yearning for my dog as he/she was before aging	○	○	○	○	○
I feel that life is empty and meaningless without my dog being healthy	○	○	○	○	○
I am bitter over my dog's aging	○	○	○	○	○
I think about my dog's aging so much that it can be hard for me to concentrate on anything else or do the things I normally do	○	○	○	○	○

Please answer the following questions about yourself.

Please select the highest level of education you have completed:

▼ High school/GED ... Other

Your current employment status:

▼ Employed full time ... Prefer to not say

Do you work:

▼ Mostly/all the time at home ... Prefer to not say

What is your current living situation?

▼ Live alone ... Prefer to not say

*

Your age:

____

How do you identify yourself?

▼ Female ... Prefer to not say

How do you identify yourself?

▼ African American/black ... Prefer to not say

Please share one story or example of something that stands out with you about living with an aging dog. It can be something positive or negative and please describe in as much detail as you are able.
